# Supplementary material for: Patterns of Intron Gain and Loss in Fungi
Source: PLoS Biol. 2004 Nov 30;2(12):e422. doi: 10.1371/journal.pbio.0020422 (PMC532390; doi:10.1371/journal.pbio.0020422)
Supplement: Table S1 — Also available at http://genes.mit.edu/NielsenEtAl/. (4.3 MB ZIP). [file pbio.0020422.st001.zip › NielsenEtAl/html/307.html]

AN6224.1.NCU05312.1.MG06076.1.FG05313.1


```
 CLUSTAL W (1.82) Multiple Sequence Alignments - Introns Inserted


Sequence 1: NCU05312.1	221 aa
Sequence 2: MG06076.1	195 aa
Sequence 3: FG05313.1	201 aa
Sequence 4: AN6224.1	200 aa
Alignment Length: 223 aa
Number Identitical Residues: 88 aa
Alignment Score (without introns) 4542


MG06076.1 	MLLEEDPAT~LIRATVQNFAVTPDKAALKRLQDSTAVLNQARELRFREAESSLKK1LSRQ
NCU05312.1	MLLEEDPST0LIAHTTQNFNITPDRHAVSRVAESLSTLQQARDLRLRESETNLKK~LART
FG05313.1 	MLLSEEPAT0LIHHTIENFNIAPDKTAVARVTESLATLQQARDLRVREAESSLKK~LSRQ
AN6224.1  	MLLDENPST0LIHHTIGNFNIAPDKQAVSRINDSLATLQQSRELRMREAESSLRK~LSRH
          	***.*:*:* **  *  ** ::**: *: *: :* :.*:*:*:**.**:*:.*:* *:* 

MG06076.1 	LNTVQSQYEELTSQHSSAAHASEMARLDTQKFRTAKAASDLEVETERLSSQAADLAARLQ
NCU05312.1	LNTLQSQHQEVTSSHSSAEHASLISRLDTQKFRVAKNVSDLEMETERLQTQLAELQARLQ
FG05313.1 	LATHTSRHDDLVTSHSSADHASNIARLDTLKFRTAKAAADAETDAERLALTAADLKARLR
AN6224.1  	LQSLSTQHEEAVAAHDASKHAAAMVELDTKKFRIAKAASELEIESERLEGELEMLKERLA
          	* :  ::::: .: *.:: **: : .*** *** ** .:: * ::***      *  ** 

MG06076.1 	ELELQGVEG-EPSAAD---------------------PVEDEVL~LRLKVYRSLGIELER
NCU05312.1	ELELQGVDGGDASAAGGATSTGENNGNGSAGNGGQVRSVEDEVL~LRLKVYRSLGIEIER
FG05313.1 	ELELQGVEGDAAANAR------------------RRDPVDDEVL~LRLKVYRSLGIDIER
AN6224.1  	DLEAQGLEGDEATRRE--------------------READDAIL2LRLKIYRALGIDIEP
          	:** **::*. .:                         .:* :* ****:**:***::* 

MG06076.1 	E---GEEWSRAVIRNDRKGDVHVVNMDK-KFSRFFYANYFWSTL--
NCU05312.1	EEQDGGEFTRAVVRNDRRGDVCVVNVDRHRFSRFFYANYFWQTL--
FG05313.1 	DER-DGEWSKAVIRNDRKGDVHVVNMDK-KFSRFFYANYFWQTL--
AN6224.1  	DE--AGNFTKAVIRNSRKGDVHVVNLDP-KFSRFFYANYFWSTMQG
          	:.    ::::**:**.*:*** ***:*  :***********.*:..
```
